# Darunavir Nanoformulation Suppresses HIV Pathogenesis in Macrophages and Improves Drug Delivery to the Brain in Mice

**DOI:** 10.3390/pharmaceutics16040555

**Published:** 2024-04-19

**Authors:** Lina Zhou, Sandip Godse, Namita Sinha, Sunitha Kodidela, Udai Singh, Santosh Kumar

**Affiliations:** Department of Pharmaceutical Sciences, University of Tennessee Health Science Center, 881 Madison Ave., Memphis, TN 38163, USAsgodse@uthsc.edu (S.G.); usingh1@uthsc.edu (U.S.)

**Keywords:** nanoparticle, BBB, HIV

## Abstract

Although antiretroviral therapy (ART) can suppress peripheral HIV, patients still suffer from neuroHIV due to insufficient levels of ART drugs in the brain. Hence, this study focuses on developing a poly lactic-co-glycolic acid (PLGA) nanoparticle-based ART drug delivery system for darunavir (DRV) using an intranasal route that can overcome the limitation of drug metabolic stability and blood–brain barrier (BBB) permeability. The physicochemical properties of PLGA-DRV were characterized. The results indicated that PLGA-DRV formulation inhibits HIV replication in U1 macrophages directly and in the presence of the BBB without inducing cytotoxicity. However, the PLGA-DRV did not inhibit HIV replication more than DRV alone. Notably, the total antioxidant capacity remained unchanged upon treatment with both DRV or PLGA-DRV in U1 cells. Compared to DRV alone, PLGA-DRV further decreased reactive oxygen species, suggesting a decrease in oxidative stress by the formulation. Oxidative stress is generally increased by HIV infection, leading to increased inflammation. Although the PLGA-DRV formulation did not further reduce the inflammatory response, the formulation did not provoke an inflammatory response in HIV-infected U1 macrophages. As expected, in vitro experiments showed higher DRV permeability by PLGA-DRV than DRV alone to U1 macrophages. Importantly, in vivo experiments, especially using intranasal administration of PLGA-DRV in wild-type mice, demonstrated a significant increase in the brain-to-plasma ratio of DRV compared to the free DRV. Overall, findings from this study attest to the potential of the PLGA-DRV nanoformulation in reducing HIV pathogenesis in macrophages and enhancing drug delivery to the brain, offering a promising avenue for treating HIV-related neurological disorders.

## 1. Introduction

HIV can enter the brain as early as day 8 of HIV infection [1]. The replication of HIV in perivascular macrophages and microglia activates the innate immune response in the brain, such as the production of inflammatory factors, including cytokines and chemokines, and oxidative stress [2,3]. In addition, astrocytes facilitate a low replication level of HIV, enabling the virus to persist and establish a latent infection in the central nervous system [4]. The advent of antiretroviral therapy (ART) has been a pivotal milestone in the management of HIV infection, drastically reducing its associated morbidity [5]. However, a significant challenge in this clinical success story is the increasing prevalence of HIV-associated neurocognitive disorders (HAND) [6,7]. A primary factor contributing to HAND is the limited ability of ART drugs to penetrate the blood–brain barrier (BBB) [8,9]. The BBB, a critical structure maintaining the brain’s homeostasis, features tight junctions and drug efflux transporters, such as multidrug-resistance protein 1 (MRP1) and P-glycoprotein (P-gp) along with cytochrome P450 (CYP) enzymes, which significantly restrict the permeability and bioavailability of ART drugs to the brain [10,11,12,13].

Maintaining an appropriate concentration of ART drugs is crucial for effectively suppressing HIV in brain reservoirs [14,15]. However, the BBB effectively shields the CNS from many therapeutic agents, including ART drugs, thus limiting their effectiveness against HIV reservoirs within the brain [15,16]. While some ART drugs have demonstrated the ability to cross the BBB, their brain concentrations are often subtherapeutic, posing a significant challenge in achieving effective HAND management [17,18]. Therefore, innovation of ART formulations with improved BBB permeability and efficacy without change in safety profile is needed. In our previous study, we have shown that poly lactic-co-glycolic acid (PLGA)-encapsulated elvitegravir (EVG) nanoparticles (NPs) can cross the BBB in vitro and in vivo and reduce HIV replication in the brains of mice [19,20].

The mechanism of drug delivery in the brain facilitated by PLGA NPs has been studied in various fields [21]. PLGA NPs can penetrate through the BBB by passive diffusion, which is limited by the size and lipophilicity of PLGA NPs. On the other hand, PLGA NPs can enter the brain via active transcytosis and endocytosis, which is usually achieved by modifying the structure of PLGA NPs to endow the target delivery [22,23]. In our previous study, we observed that the PLGA NPs encapsulated with EVG cross the BBB mainly through clathrin-mediated endocytosis in vitro [20,24]. In this study, we selected another relevant ART drug, darunavir (DRV), a protease inhibitor, to develop a PLGA-based delivery system. DRV demonstrates relatively better BBB permeability and lower neurotoxicity than other ART drugs, which makes DRV a suitable candidate for CNS-targeted therapy [25,26]. The objective of this study is to develop a PLGA-DRV nanoformulation that shows improved HIV pathogenesis without showing cytotoxicity or immune response compared to DRV alone in macrophages and enhances DRV permeability in the brain.

## 2. Materials and Methods

### 2.1. Materials

DRV (D193500) was purchased from Toronto Research Chemicals, Inc. (North York, ON, Canada). PLGA (50:50 lactide–glycolide ratio, Mw: 31,000–50,000, ester-terminated) was brought from Birmingham Polymers (Pelham, AL, USA). The following items were obtained from Sigma-Aldrich Co. (St. Louis, MO, USA): poloxamer 188 (pluronic F-68) (P1300, Mw: 8350), poly(vinyl alcohol) (PVA) (363138, Mw: 30,000–70,000), poly(L-lysine) (PLL) (Mw: 30,000–70,000). Sterile phosphate-buffered saline (PBS) (10100-031) was obtained from Gibco (Dublin, Ireland). L-glutamine, penicillin–streptomycin solution, LC/MS-grade acetonitrile (A955), formic acid (AC270480010), BD PrecisionGlide 25G needle (14-826-49), and BD 1 Ml TB syringe (14-826-88) were obtained from Fisher Scientific (Pittsburgh, PA, USA). Roswell Park Memorial Institute (RPMI) 1640 media was bought from Corning Inc. (Tewksbury, MA, USA). Fetal bovine serum (FBS) was obtained from Atlanta Biologicals (Atlanta, GA, USA). Dulbecco’s modified Eagle’s medium (DMEM) was obtained from American Type Culture Collection. The immortalized mouse brain endothelial cells (bEnd.3, CRL-2299) and mouse astrocytes (C8-D1A, CRL-2541) were purchased from American Type Culture Collection (Manassas, VA, USA). UranyLess (22409) was from Electron Microscopy Science (Hatfield, PA, USA). Constitutively HIV-infected (U1) cell lines were obtained from NIH AIDS Reagent Program (Germantown, MD, USA). Paraformaldehyde (J19943.K2) was purchased from Thermo Fisher Scientific (Waltham, MA, USA).

### 2.2. Preparation of PLGA-DRV

PLGA-encapsulated DRV nanoparticle (PLGA-DRV) formulation and control PLGA were prepared by nano-precipitation technique as described [27]. In brief, a solution containing 45 mg of PLGA and 5 mg of DRV was prepared by dissolving these components in 4 mL of acetone, resulting in a homogeneous PLGA-DRV solution. This solution was then added dropwise into 10 mL of a 1% PVA aqueous solution using a magnetic stirrer set at 400 rpm. Following the complete evaporation of acetone, which took approximately 3 h, 10 mg of PLL was dissolved in 1 mL of water, and 50 mg of the pluronic polymer F-68 was dissolved in 4 mL of water. Both the PLL and F-68 solutions were then added to the nanoparticle suspension and stirred at room temperature for about 12 h to ensure the full evaporation of acetone. Larger, non-uniform aggregates comprising PLGA, DRV, PVA, and PLL were eliminated through centrifugation at 1000 rpm for 10 min. The supernatant was collected as the uniform PLGA-DRV nanoparticle formulation was then stored at 4 °C for future use.

### 2.3. Characterization of PLGA Nanoparticles

The average particle size and zeta potential of the prepared PLGA-DRV nanoparticles were determined by the dynamic light scattering (DLS) using Zetasizer (Nano ZS, Malvern Instruments, Malvern, UK) following the company’s protocol. To prevent multi-scattering phenomena, 50 µL of PLGA-DRV formulation was diluted in 850 µL of 1× PBS (0.22 µm filtered) for size and zeta potential analyses. Zeta potential calculation was performed by Zetasizer software (Version 7.11). Size and zeta potential measurements were conducted in triplicate, and the results were reported as mean ± SD (standard deviation). To prepare the sample for transmission electron microscope (TEM), the PLGA-DRV samples were diluted (1:500) in diH_2_O (0.22 µm filtered). Ten microliters of samples were added and dried on the grid and fixed with UranyLess. The morphology of PLGA-DRV nanoparticles was confirmed by TEM (JEOL-2000EX, Tokyo, Japan).

### 2.4. Encapsulation Efficiency and Drug Loading

PLGA-DRV formulation (1 mL) was mixed with 4 mL of acetone for at least 2 h at room temperature to extract DRV for measurement of encapsulation efficiency. To determine the drug loading capacity, PLGA-DRV formulation (1 mL) was lyophilized using the Labconco Freeze Dry System overnight (−48 °C, 133 × 10^−3^ mBar; Labconco, Kansas City, MO, USA). Lyophilized PLGA-DRV formulation was reconstituted in 4 mL acetone to extract DRV for the drug loading. To ensure thorough extraction of DRV from the nanoparticles, the acetone solution was placed on a shaker (Corning, LSE Digital Microplate Shaker, Tewksbury, MA, USA) and gently shaken for 24 h at 80 rpm at room temperature to completely extract DRV from the nanoparticles. The supernatant was collected and diluted (1:300) in acetonitrile to measure the concentration in LC-MS/MS.

$$Encapsulation\;efficiency\;(\%)=\frac{Weight\;of\;the\;drug\;in\;nanoparticles}{Weight\;of\;the\;feeding\;drugs}\times 100$$

$$Drug\;loading\;(\%)=\frac{Weight\;of\;the\;drug\;in\;nanoparticles}{Weight\;of\;the\;nanoparticles}\times 100$$

### 2.5. DRV Quantification by LC-MS/MS

DRV concentrations in formulation, cell lysates, mouse plasma, and tissue samples were measured using the established LC-MS/MS method we have previously detailed [28,29]. Prior to collection for in vitro study, differentiated U1 cells, also known as U1 macrophages, underwent a washing process with PBS and were then harvested using RIPA buffer. Quantification of DRV and the internal standard RTV (at a concentration of 50 ng/mL) was conducted using an AB SCIEX Triple Quad 5500 tandem mass spectrometer, which featured an electron spray ionization source operating in positive mode. The separation of these compounds was achieved through a liquid chromatography system, specifically the LC-20AD XR model from Shimadzu, MD. Data acquisition and analysis were carried out in multiple-reaction monitoring (MRM) mode using MultiQuant^®^ software (Version 3.0.2) by AB Sciex (Foster City, CA, USA). To mitigate matrix effects, calibration curves were constructed using blank control samples, such as plasma or tissue homogenates, tailored to the types of samples being analyzed. These controls showed no significant interference. All samples underwent centrifugation at 10,000 rpm using a Centrifuge SORVALL LEGEND X1R by Thermo Scientific, USA, after which the supernatant was further processed for LC-MS/MS analysis.

### 2.6. In Vitro Drug Release

PLGA-DRV nanoparticles were studied for in vitro release using the procedure described in our previous publication with few modifications [19,20,24]. Briefly, the in vitro drug release experiment was conducted using a Float-A-Lyzer^®^ dialysis device (Repligen Corporation, Rancho Dominguez, CA, USA), which has a molecular weight cut-off range between 8 and 10 kDa (Sigma-Aldrich Co., catalog number Z726508). A total of 5 mL of the PLGA-DRV NPs formulation was placed inside the dialysis device, which was then immersed in 80 mL of cell culture medium. This setup was maintained at 37 °C with gentle stirring. Samples of 500 µL from the surrounding medium were collected at intervals of 15 and 30 min, and 1, 3, 6, 9, 12, and 24 h for the purpose of measuring DRV concentrations using the LC-MS/MS technique. After each sample collection, an equivalent volume of 500 µL of fresh medium was replenished to maintain the volume constant throughout the experiment.

### 2.7. Cell Culturing

For direct treatment, we cultured the U1 cells in RPMI 1640 media supplemented with 10% fetal bovine serum (FBS) and 1% L-glutamine. We seeded 0.4 million U1 cells in 1 mL of media containing 100 nM phorbol 12-myristate 13-acetate (PMA) in each well of a 12-well plate for differentiation of U1 cells to U1 macrophages. After 72 h of differentiation, the media was aspirated, and the cells were washed with PBS before adding fresh media to the differentiated cells. The cells were then incubated for 3–4 h before starting the treatment.

For the in vitro BBB model, mouse brain endothelial cells (bEnd.3) and mouse astrocytes (C8-D1A) were grown in complete media, which was composed of DMEM supplemented with 10% fetal bovine serum and 1% penicillin–streptomycin solution. Cultivation occurred in T75 flasks maintained at 37 °C in a 5% CO_2_ humidified atmosphere. To assemble this model, Transwell^®^-COL collagen-coated inserts with 0.4 μm pore size made of polytetrafluoroethylene membrane (Sigma-Aldrich) were employed, as described in previously established protocols [19,30,31]. Mouse astrocytes were initially plated at the bottom of 12-well plates at a density of 3 × 10^5^ cells/well. Following a 2–3 day period allowing for cell adhesion, mouse endothelial cells were seeded on the upper side of the Transwell^®^-COL inserts at a similar density of 3 × 10^5^ cells/well. These inserts were then integrated into the 12-well plates with the astrocytes. The resulting in vitro BBB model was cultured for an additional 5 days until they reached approximately 90% confluency. On day 3 of the BBB cell culturing, U1 cells were seeded in separate 12-well plates for differentiation, as mentioned before. On day 5, the inserts carrying bEnd.3 cells were transferred to the 12-well plate with the U1 cells.

The differentiated U1 macrophages were subjected to different treatment conditions. This included a control group treated with DMSO, as well as experimental groups treated with DRV (6 µg/mL) and PLGA-DRV (containing 6 µg/mL DRV). These concentrations, which are near-physiological, were chosen based on our previous study [28]. The cells were exposed to the respective treatments for a defined period as per the treatment protocol of each assay. After the treatment duration, the U1 macrophages were harvested for further analysis. The cells were collected and processed for downstream experiments as per the specific requirements of each assay.

### 2.8. LDH Cell Viability Assay

Cytotoxicity levels were assessed using the Pierce Lactate Dehydrogenase (LDH) Cytotoxicity Assay Kit from Thermo Fisher Scientific (Grand Island, NY, USA), adhering to the guidelines provided by the manufacturer. The presence of LDH in the cell culture media serves as a marker for cell damage and lysis. Specifically, 50 μL of the sampled media was combined with 50 μL of the LDH assay reagent in a 96-well plate. This mixture was then incubated at ambient temperature for 30 min before the reaction was halted by the addition of an LDH stop solution. The absorbance was recorded at wavelengths of 490 nm and 680 nm by a microplate reader (Cytation™ 5 Cell Imaging Multi-Mode Reader, BioTek, VT, USA). The final measurement was obtained by subtracting the 680 nm absorbance value from the 490 nm absorbance value, with higher differences indicating greater cytotoxicity.

Corrected LDH Activity = A_490_ − A_680_

Relative LDH Activity = (Corrected LDH Activity of Experimental Group)/(Corrected LDH Activity of Control Group) × 100%

### 2.9. HIV Type 1 p24 ELISA

The HIV-1 p24 antigen levels in U1 cell supernatants were determined using a Zeptometrix Corporation ELISA Kit (Buffalo, NY, USA), adhering to the provided instructions. This process involved adding the samples to microwells coated with a monoclonal antibody specific to the HIV-1 p24 antigen. These samples underwent a sequence of incubations: first with a biotin-labeled human antibody against HIV-1 for an hour, then with streptavidin-horseradish peroxidase conjugate for 30 min at 37 °C, and finally with a tetramethylbenzidine substrate for 30 min in darkness. The optical density at 450 nm for each well was measured and the absorbance was used to calculate the p24 antigen levels in picograms per milliliter (pg/mL). The standard curve for p24 was used to calculate the p24 levels in our samples. The results were expressed as a percentage compared to the p24 levels in control wells.

### 2.10. Total Antioxidant Capacity (TAC)

The antioxidant capacity of U1 macrophages after treatments was determined using the Total Antioxidant Capacity Assay (TAC) Kit (Cell Biolabs, San Diego, CA, USA). This assay was carried out adhering to the guidelines specified by the manufacturer. This assay determines the antioxidant capacity by assessing the copper-reducing equivalents (CREs) present in the samples. The findings are expressed in μM of CREs, reflecting the overall antioxidant capacity of the samples.

### 2.11. Quantification of Intracellular Reactive Oxygen Species (ROS) with Fluorescence-Based Assay

To quantify the ROS level, we performed flow cytometry analysis using fluorescence dye chloromethyl derivative of 2′,7′-Dichlorodihydrofluorescein diacetate (CM-H2DCFDA) (Thermo Fisher Scientific) as described before [32]. Following a thorough wash of the treated cells with 500 µL of PBS, they were resuspended in a solution of 5 μM CM-H2DCFDA mixed in PBS with 1% FBS and subsequently incubated in darkness at room temperature for 45 min. Post-incubation, the cells underwent another washing step and were then resuspended in 300 μL of PBS with 1% FBS. The cells were fixed with 2% paraformaldehyde for flow cytometry analysis. The ROS generated from the cells were detected and quantified using the NovoExpress software (Version 1.5.6).

### 2.12. Detection of Cytokines and Chemokines

We measured the levels of various cytokines and chemokines, including pro-inflammatory cytokines IL-1β, TNF-α, IL-6, and IL-18; anti-inflammatory chemokines IL-1RA, IL-8, and IL-10; and chemokines MCP-1 and RANTES in the culture media of U1 macrophages. The Human Custom Procartaplex 9-plex (Invitrogen, Thermo Fisher Scientific, Grand Island, NY, USA) was used following the manufacturer’s protocol as previously described [33]. In summary, the procedure involved mixing samples, standards, and magnetic beads within a 96-well ELISA plate, which was then thoroughly agitated on a plate shaker for one hour at ambient temperature, followed by an incubation period overnight at 4 °C. Post-incubation, the beads were washed before each addition of the detection antibody, streptavidin-PE, and reading buffer. The quantification of cytokines and chemokines, expressed in pg/mL, was performed utilizing the Magpix system (Luminex, Austin, TX, USA), with data interpretation conducted through the xPONENT^®^ software (Version 4.2).

### 2.13. Animal Studies

Ten twelve-week-old male and female Balb/c mice were purchased from Jackson Laboratory (Bar Harbor, MA, USA) and were acclimated to the animal facility for at least 7 days. Five mice per cage were housed in a sterile room with 12/12 h light–dark cycles. Temperature and humidity were maintained at a constant level in the room. There was free access to food and water. Detailed information for dosing in Balb/c mice can be found in our previous study [29]. A 2.5 mg/kg dosage of DRV or PLGA-DRV NPs was given via intranasal (IN) and intravenous (IV). For the IN group, the minimum concentration of DRV is 1.25 mg/mL to ensure that the dosing volume for each mouse is less than 2 µL per gram of mice. Given the constraints on the EE (%) of DRV in PLGA, we selected a dosage of 2.5 mg/kg, representing the highest dose achievable within the scope of this study. Animal studies were performed according to The University of Tennessee Health Science Center Institutional Animal Care and Use Committee (UTHSC-IACUC) protocol. All methods were carried out in accordance with relevant guidelines and regulations. All experimental protocols involving the use of laboratory animals were approved by the UTHSC Institutional Animal Care and Use Committee (IACUC).

## 3. Results

### 3.1. Characterization of PLGA-DRV

**Figure 1:**
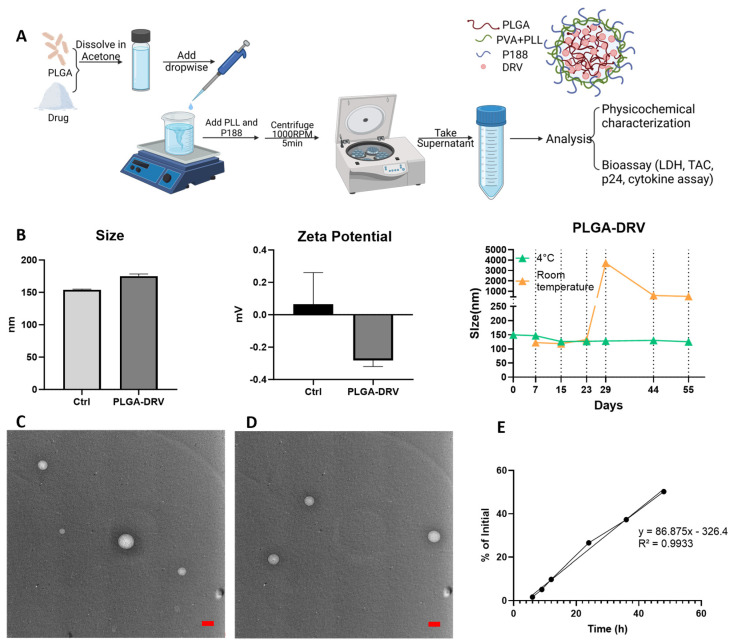
(**A**) Schematic diagram of the preparation of PLGA-DRV NPs and hypothetical structure of PLGA-DRV NPs. (**B**) Size and zeta potential profile from DLS measurements: Ctrl 154.1 ± 0.93 nm, DRV 175.1 ± 3.30 nm. Encapsulation efficiency was DRV 84.19% ± 6.60; mean ± SD was analyzed from 3 measurements. Drug loading was 1.94%. Zeta potential: Ctrl 0.065 ± 0.195 mV, DRV −0.283 ± 0.037 mV. Size of PLGA-DRV was measured over ~8 weeks at room temperature and 4 °C. Transmission electron microscopy (TEM) images of (**C**) PLGA Ctrl NPs showed a size of 138.9 nm and (**D**) PLGA-DRV NPs showed a size of 111.1 nm. Red scale bar 100 nm. (**E**) In vitro drug release profile of PLGA-DRV NPs.

The PLGA-DRV NP formulation was prepared with an oil-in-water (*o*/*w*) system using nanoprecipitation. Figure 1A illustrates the formulation preparation and a proposed structure for PLGA-DRV NPs. In this structure, PLGA forms the primary polymer core encapsulating the DRV molecule. PVA was added as a stabilizer. Poloxamer 188 acts as a stabilizer, and it also enhances brain penetration. PLL imparts a mild positive charge, making the PLGA NP less negative, which aids cellular uptake, biodegradation, and controlled release of drugs [34]. The size of freshly made PLGA NPs was determined by DLS. The blank PLGA NPs demonstrated a size of 154.1 ± 0.93 nm with a zeta potential of 0.065 ± 0.195 mV, and the DRV-loaded NPs were 175.1 ± 3.30 nm with a zeta potential of −0.283 ± 0.037 mV (Figure 1B). The size of PLGA-DRV was measured at room temperature and at 4 °C over 8 weeks. At room temperature, the PLGA-DRV maintains a size comparable to the fresh formulation for up to four weeks. At 4 °C, the particle size remains similar to that of a freshly prepared batch for the entire duration of the eight-week measurement period.

The TEM images of PLGA nanoparticles are shown in Figure 1C (PLGA NPs without loading DRV) and Figure 1D (PLGA NPs with DRV loading). The morphology of PLGA Ctrl NPs and PLGA NPs with DRV loading were observed. The size of PLGA NPs was roughly estimated to be below 200 nm, but a more accurate value cannot be obtained through visual judgment using TEM alone due to the limited number of particles. The encapsulation efficiency (EE%) of the PLGA-DRV NPs was 84.19% ± 6.60 with a drug loading of 1.94%. Although the drug loading percent appears to be low, this provided the most optimal EE% for PLGA-DRV nanoformulation. If needed, we will further optimize the PLGA-DRV formulation that creates an optimal balance of both drug loading percent and EE%. The drug encapsulation increased the size of NPs, which aligned with our previous results [7]. Figure 1E shows the in vitro release profile of PLGA-DRV NPs in cell culture media, which indicated a zero-order release with r^2^ of 0.99 within 48 h. Approximately 25% and 50% percent of cumulative DRV compared to freshly prepared formulation were released at 24 h and 48 h, respectively, suggesting a potential application in controlled release.

### 3.2. Effect of PLGA NPs on Cytotoxicity in U1 Macrophages in the Absence and Presence of In Vitro BBB Model

**Figure 2:**
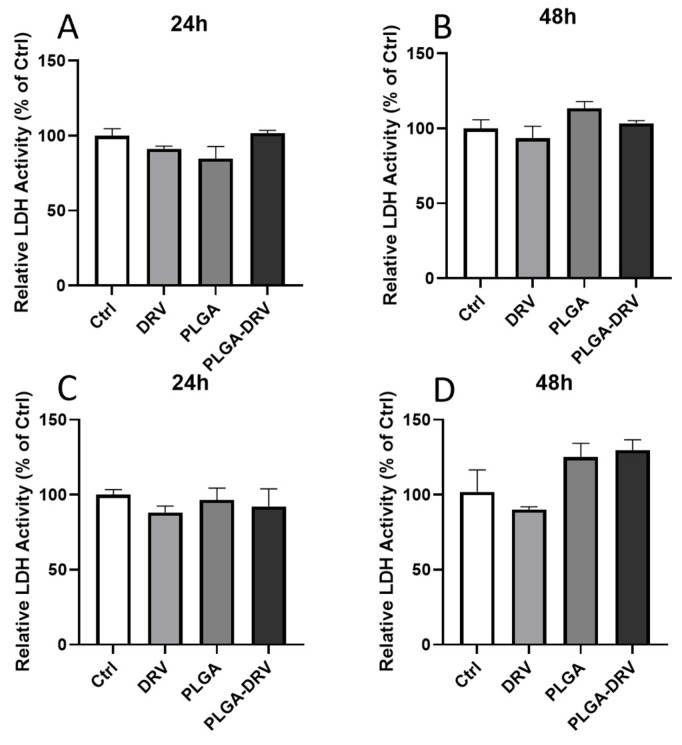
Cytotoxicity of DRV and PLGA-DRV. (**A**,**B**) LDH activity of U1 macrophages after 24 h and 48 h treatment of 6 µg/mL DRV or PLGA-DRV. (**C**,**D**) LDH activity of U1 macrophages in the model with a BBB after 24 h and 48 h treatment of 6 µg/mL DRV or PLGA-DRV. Mean ± SEM was graphed from 3 measurements. One-way ANOVA test was used for data analysis.

LDH cytotoxicity assay was performed to determine whether the treatment with PLGA-DRV causes cell death. When cells are damaged or undergo cytotoxicity, they release LDH into the culture medium, making it a useful indicator of cell membrane integrity and cell death. U1 macrophages, a monocyte cell line infected with HIV, were treated with control, DRV, PLGA, and PLGA-DRV for 24 h and 48 h in the absence and presence of in vitro BBB model (Figure 2). At 48 h, the LDH activity in PLGA and PLGA-DRV seemed higher than the control; however, there were no significant changes among all groups determined from the one-way ANOVA test, as shown in Figure 2. The results indicated that the DRV, PLGA NPs, and PLGA-DRV did not cause significant cytotoxicity in U1 macrophages directly or in the presence of a BBB up to 48 h.

### 3.3. Effect of PLGA NPs on Antioxidant Capacity and Oxidative Stress in U1 Macrophages in the Absence and Presence of In Vitro BBB Model

**Figure 3:**
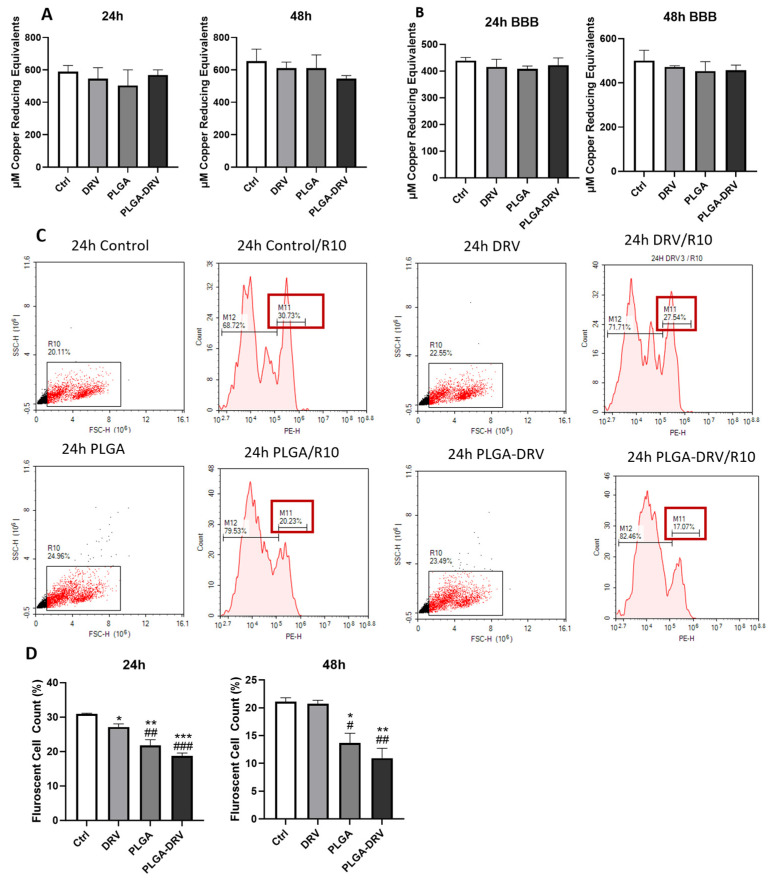
(**A**) U1 cells were differentiated for 72 h and treated with 6 μg/mL DRV or PLGA-DRV for 24 h and 48 h and TAC was measured. (**B**) The same treatment was performed in the presence of a BBB for 24 h and 48 h, and TAC assay was measured. Results were normalized based on protein level of U1 macrophages. Mean ± SEM were graphed from 3 measurements. (**C**,**D**) ROS activity was measured in the direct treatment of U1 *w*/*o* BBB by flow cytometry using CM-DCFDA dye and excitation/emission at 495/519 nm. Red box indicates the percentage of fluorescent cell count. Data were quantified using florescent cell count that was measured in %. Results are expressed as means ± SEM of *n* = 3 experiments. One-way ANOVA test was used for data analysis; * in comparison with untreated control, # in comparison with DRV; * or # *p* < 0.05, ** or ## *p*< 0.01, *** or ### *p* < 0.001.

Given that the treatment of PLGA-DRV did not have cytotoxicity on U1 with and without a BBB, the TAC was measured. The TAC was indicated by the concentration of copper-reducing equivalents. As shown in Figure 3A,B, the PLGA NPs did not alter the TAC in the U1 macrophages either in direct treatment with U1 macrophages or in the presence of a BBB.

The oxidative stress was assessed by determining the ROS level by flow cytometry analysis (Figure 3C,D). We have gated the overall population that expressed differential levels of ROS. After 24 h treatment, DRV alone significantly reduced the ROS level as compared to control (* *p* < 0.05). Importantly, PLGA-DRV also significantly reduced the ROS level in these overall populations (*p* < 0.001) at 24 h, which was reduced more than DRV alone (* *p* < 0.05). After 48 h treatment, DRV did not retain the reduced ROS level, but PLGA-DRV did retain the reduced ROS level (** *p* < 0.01). Overall, the results suggest that PLGA-DRV is more effective in reducing oxidative stress in the overall cell population than DRV alone, at least at the early time point.

### 3.4. Effect of PLGA NPs on Cytokines and Chemokines in U1 Macrophages in the Absence and Presence of In Vitro BBB Model

**Figure 4:**
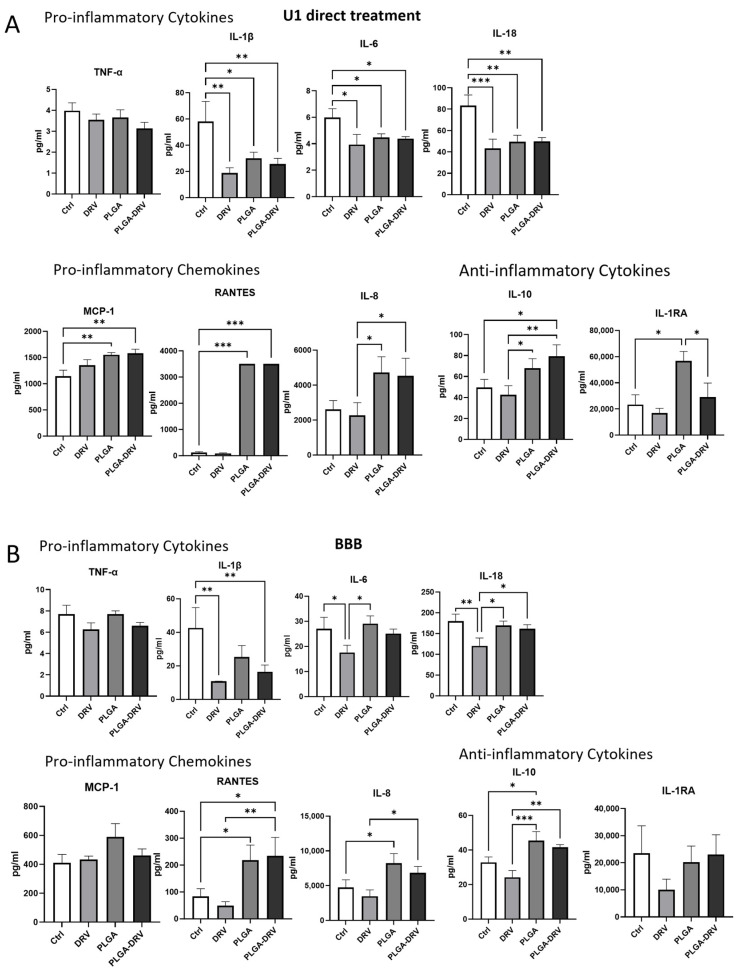
Multiplex ELISA of cytokines and chemokines assay in U1 macrophages upon exposure to DRV, PLGA, and PLGA-DRV for 48 h (**A**) direct treatment and (**B**) in the presence of in vitro BBB. Results are expressed as means ± SD. One-way ANOVA test was used for data analysis; * *p* < 0.05, ** *p* < 0.01, *** *p* < 0.001.

Monocytes and macrophages are key players in innate immunity, and they respond to HIV infection by releasing pro-inflammatory cytokines and chemokines [35]. Therefore, we evaluated some of the cytokines and chemokines related to HIV infection in U1 macrophages after treatment with DRV and PLGA-DRV for 48 h with direct treatment and in the presence of in vitro BBB (Figure 4). 

The observed patterns in cytokine and chemokine secretion by U1 macrophages were consistent, regardless of the presence of an in vitro BBB. Specifically, in the direct treatment scenario (Figure 4A), there was a significant reduction in the levels of pro-inflammatory cytokines IL-1β, IL-6, and IL-18 following treatment with DRV and PLGA-DRV as compared to control. However, the levels of these cytokines were not further decreased in PLAGA-DRV as compared to DRV alone. Unexpectedly, PLGA alone also showed a significant reduction in these pro-inflammatory cytokines. On the other hand, IL-8 pro-inflammatory chemokine level was increased in PLGA and PLGA-DRV groups. None of the treatment groups substantially lowered the level of TNF-α cytokines in the U1 macrophages as compared to control. Additionally, PLGA NPs significantly elevated the production of pro-inflammatory chemokines MCP-1 and RANTES. However, there was no further increase in these pro-inflammatory chemokines when treated with PLGA-DRV as compared to PLGA. It was also observed that PLGA alone significantly raised the levels of anti-inflammatory cytokines IL-10 and IL-1RA, but PLGA-DRV did not further increase their levels. Instead, compared to PLGA alone, PLGA-DRV decreased the levels of IL-1RA.

When the BBB model was introduced, a similar pattern of changes in cytokine and chemokine levels was noted. However, due to larger standard deviations and relatively smaller changes in the BBB experimental setup compared to the direct treatments, the changes in pro-inflammatory cytokines IL-1β, IL-6, and IL-18, and chemokines RANTES and MCP-1, especially with the PLGA-DRV formulation, were not as pronounced as those seen with direct treatment. Nonetheless, the changes in IL-8 were similar in the BBB model as compared to direct treatment, although in direct treatment, due to deviation, the change in PLGA was not significant as compared to the control. For the anti-inflammatory cytokine IL-10, PLGA NPs demonstrated a significant increase (*p* < 0.05), and PLGA-DRV further amplified this effect compared to DRV alone (*p* < 0.01). However, with IL-1RA anti-inflammatory cytokine, unlike direct treatment, there was no significant change with any groups in the BBB model.

Overall, the results suggest that compared to DRV alone, PLGA-DRV directly or in the presence of an in vitro BBB does not improve immune response in U1 macrophages either by reducing pro-inflammatory cytokines/chemokines and/or increasing anti-inflammatory cytokines. However, the findings that the PLGA-DRV formulation does not cause a further inflammatory response, compared to DRV alone, is important in the context of using this nanoformulation in animal models.

### 3.5. Intracellular DRV Concentration

In our previous study, we observed that PLGA-EVG improves the cellular uptake of the formulation in U1 macrophages [19]. So, in this study, we also performed an experiment to determine whether the intercellular concentration of DRV was improved by the PLGA formulation.

**Figure 5:**
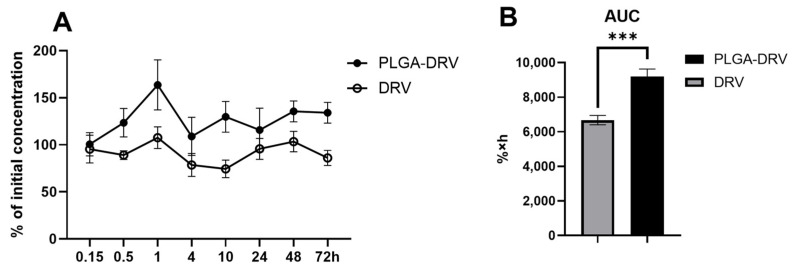
(**A**) Intracellular concentration profile of DRV and PLGA-DRV. The U1 cells were treated with 6 μg/mL DRV or PLGA DRV for 0.15, 0.5, 1, 4, 10, 24, 48, and 72 h. The intracellular concentration of DRV was measured by LC-MS/MS. (**B**) Area under the curve (% × hours(h)) of DRV and PLGA-DRV nanoparticles. *T*-test was used for data analysis; *** indicates *p* < 0.001 compared to treatment with DRV alone. Mean ± SEM values were graphed (*n* = 6).

The U1 macrophages were treated with control, DRV, PLGA, and PLGA-DRV for 0.15, 0.5, 1, 4, 10, 24, 48, and 72 h. To evaluate the intracellular concentration of DRV, we performed an LC/MS-MS method using RTV as an internal control. The standard curve was achieved over the range of 0.5–2000 ng/mL using a weighting factor of 1/x^2^ regression (r^2^ = 0.99). The average concentration of DRV at 0.15 h in corresponding treatment groups was used as the initial concentration. We plotted the percentage of the initial intracellular concentration of DRV (Figure 5A). Our results showed that PLGA-DRV maintained a higher percentage of initial DRV concentration throughout the treatment. The area under the curve (AUC) of the PLGA-DRV group was significantly (~1.4 times) higher than that of the free DRV group (Figure 5B). This observation elucidated that the encapsulation of DRV within PLGA NPs augmented both its stability and bioavailability. This enhancement in the in vitro pharmacokinetic profile suggested that PLGA-encapsulated DRV could enhance the bioavailability and efficacy of the DRV in an animal model.

### 3.6. Effect of PLGA NPs in Viral Suppression in U1 Macrophages in the Absence and Presence of In Vitro BBB

In our study, we first treated U1 macrophages with 6 µg/mL DRV and 6 µg/mL PLGA-DRV for 24 h and 48 h. The replication of HIV was assessed by measuring the concentration of the p24 protein to determine the efficacy of the viral suppression of DRV nanoformulation.

**Figure 6:**
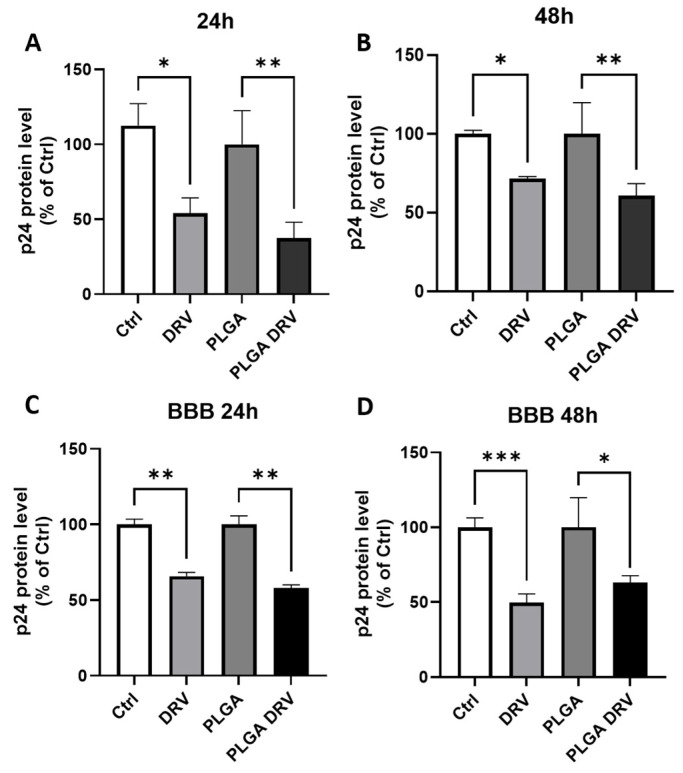
HIV-1 replication after exposure to 6 µg/mL DRV and 6 µg/mL PLGA-DRV for 24 h and 48 h. p24 level in U1 media was measured. Data were normalized by protein level of U1 and reported as percentage of control groups of free DRV and PLGA NPs. (**A**,**B**) Direct treatment in U1 macrophages for 24 h and 48 h. (**C**,**D**) Treatment in U1 macrophages in the presence of a BBB for 24 h and 48 h. Mean ± SEM were graphed from 3 measurements. * *p* < 0.05, ** *p* < 0.01, *** *p* < 0.001.

In the direct treatment of DRV and PLGA-DRV to U1 macrophages (Figure 6A,B), as expected, DRV led to a reduction in p24 levels by approximately 45% at 24 h and about 28% at 48 h, compared to control. The PLGA-DRV demonstrated a pattern of further reduction in p24 levels in U1 macrophages (62% at 24 h and 39% at 48 h) compared with DRV or PLGA. However, the further reduction was not statistically significant.

U1 macrophages were further subjected to the same treatment protocol as in the direct treatment methodology in the presence of the BBB model (Figure 6C,D). The findings revealed that DRV significantly reduced p24 levels across the BBB. However, the PLGA-DRV did not show a further reduction in p24 levels across the BBB.

Overall, the in vitro experiments showed the following: (1) PLGA-DRV can increase DRV concentration compared to DRV alone in U1 macrophages. (2) Compared to DRV, PLGA-DRV decreases oxidative stress. (3) PLGA-DRV does not elicit cytotoxicity in U1 macrophages. (4) Although PLGA-DRV suppresses HIV and inflammatory responses as compared to control, it does not further significantly suppress HIV replication and inflammatory responses compared to DRV alone. (5) The PLGA-DRV nanoformulation can penetrate the in vitro BBB, suppress HIV infection, and elicit an altered inflammatory response in U1 macrophages. However, PLGA-DRV does not further decrease HIV replication or inflammatory response compared to DRV alone across the BBB. Therefore, in the following studies, we performed in vivo experiments with PLGA-DRV formulation.

### 3.7. DRV Levels in Mice

**Figure 7:**
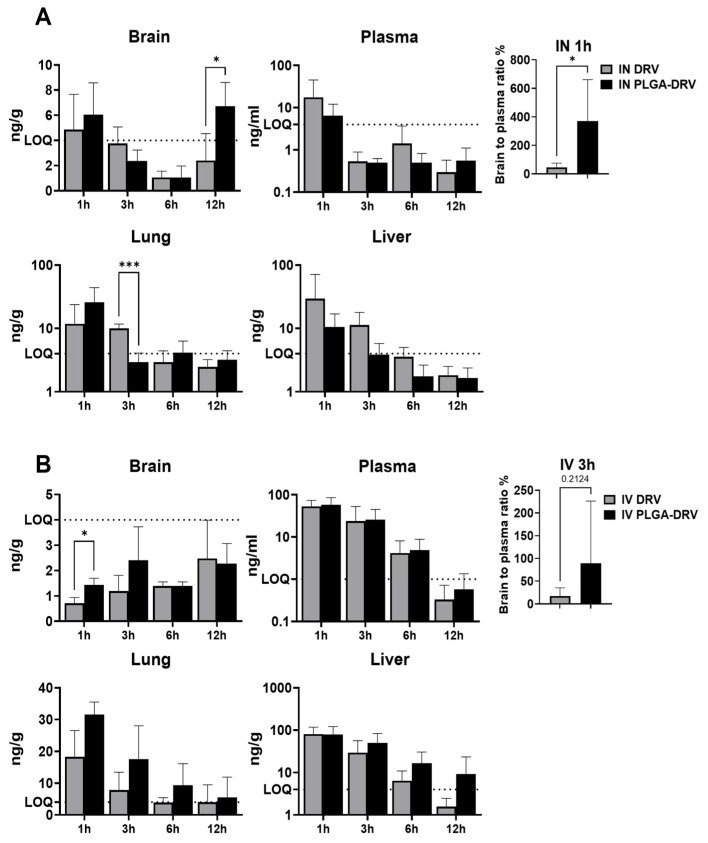
Biodistribution of 2.5 mg/kg DRV administered via (**A**) intranasal (IN) and (**B**) intravenous (IV) in Balb/c mice. DRV concentration was determined in mice brains, plasma, lungs, and livers after dosing with DRV or PLGA-DRV at 1 h, 3 h, 6 h, and 12 h. The brain-to-plasma concentration was calculated by dividing the DRV concentration in the brain by the DRV concentration in the plasma ×100%. Limit of quantification (LOQ) is indicated by the dash line. Results are expressed as means ± SD. *T*-tests were used to compare the differences between two treatment groups; * indicates *p* < 0.05, *** indicates *p* < 0.001, *n* = 6.

We evaluated whether PLGA-DRV can increase the DRV concentration in the brains of mice. In this study, both DRV and PLGA-DRV were administered to mice at a dose of 2.5 mg/kg, utilizing intranasal (IN) (Figure 7A) and intravenous (IV) routes (Figure 7B).

As expected, the IN administration showed higher brain DRV concentration in mice brains of the PLGA-DRV group at 1 h and 12 h. The DRV concentration fell under LOQ at 3 h and 6 h (Figure 7A). The plasma concentration of DRV following IN administration decreased over time in DRV and PLGA-DRV groups. Due to the LOQ of brain and plasma, brain to plasma (B/P) ratio was calculated as the ratio of brain to plasma concentration × 100% only at 1 h. PLGA-DRV showed a significant improvement in the B/P ratio compared to the DRV group through IN. In addition, via IN administration at an early time point (1 h) and a late time point (12 h), the DRV concentration in the PLGA-DRV group was markedly higher in the brain and lung while it was lower in plasma and liver. This suggests that IN could endow direct drug delivery to the brain, at least in part, via olfactory mucosa.

Compared to IN, IV administration showed a higher systematic exposure to DRV in peripheral organs (Figure 7B). The majority of the DRV concentration in the brain fell under the LOQ, and the drug concentration in brain samples may not be accurately estimated. This observation suggests that IN administration is better than IV for drug infiltration to the brain. Although not accurate, we observed a trend that drug concentration reached a peak concentration at a later time point with IV than with IN, which shows bimodal peaks due to direct absorption to olfactory mucosa and drug delivery via BBB. Further, the PLGA-DRV group showed a higher B/P ratio at 3 h in IV, though the difference was not statistically significant. Although the mean plasma concentration of DRV is similar between the PLGA-DRV group and the DRV group through the IV route, the exposure to DRV was higher in the PLGA-DRV group in the lungs and perhaps in the liver. When we compared the concentration of DRV in the same organs in IV and IN regardless of PLGA encapsulation, we found that DRV concentrations were higher in the brain throughout all time points and at 1 h in the lungs via IN, while DRV concentrations were higher in the plasma and the liver via IV. This implies that PLGA-DRV IV administration will endow a higher systemic exposure to DRV, and PLGA-DRV will further increase the exposure in the liver through IV compared to IN.

## 4. Discussion

HAND encompasses a range of cognitive impairments, from mild deficits to severe dementia, adversely impacting the quality of life of those affected [36,37]. This ongoing concern underscores a critical gap between the efficacy of current ART regimens in the CNS and peripheral system, particularly in relation to their neurological impact. DRV is an ART drug that shows certain BBB permeability; however, the drug concentration is not sufficient to suppress HIV in the brain [38,39]. HAND persists in those patients who have suboptimal DRV concentration in the brain [38,39]. Therefore, to effectively treat HIV pathogenesis in the brain, an optimal concentration of DRV can be achieved by improving its BBB permeability [25,38]. Therefore, an innovative drug delivery approach via synthetic PLGA nanoparticles is needed to enhance DRV concentrations in the HIV brain reservoirs, especially macrophages, to suppress HIV pathogenesis [40].

Recently, PLGA NPs have been studied and shown remarkable potential for various CNS diseases, such as ischemic stroke [41], Alzheimer’s disease [42], glioma [43,44], Parkinson’s disease [45,46], etc. Though a lot of research efforts have been involved in the application of PLGA NPs in drug delivery to CNS, there are limited studies on the application of PLGA NPs in HAND. We have successfully encapsulated an ART drug, EVG, in PLGA NPs and suppressed HIV replication in CNS reservoirs in vitro [19]. Latronico et al. reported that with DRV encapsulated in PLGA, the amount of DRV that crosses the BBB has significantly improved with an enhanced inhibition of matrix metalloproteinase-9 (MMP-9) expression in vitro, which is a key element involved in the progression of HAND [40].

In this study, we formulated PLGA-DRV and studied its effects on HIV pathogenesis in U1 macrophages directly and across the BBB. We also tested the ability of PLGA-DRV to enhance brain drug concentration in the animal model. The physicochemical properties of PLGA-DRV NP formulation were also characterized, which ensured its suitability for CNS delivery. In vitro studies showed the ability of PLGA-DRV to suppress HIV replication and reduce oxidative stress and inflammatory response without causing cytotoxicity. However, except for oxidative stress, the formulation did not show further improvements in suppression of HIV replication or reduction in inflammatory response. In vivo studies showed the enhanced ability of PLGA-DRV to accumulate in mice brains compared to DRV alone, especially with the IN administration. Overall, the findings attest to the potential of the PLGA-DRV in reducing HIV pathogenesis safely and effectively, offering a promising avenue for treating HIV-related neurological disorders.

The average size for the PLGA-DRV is under 200 nm, which is within the appropriate range to endow its BBB permeability [47]. We also identified that PLGA-DRV can maintain its structure at 4 °C and even at room temperature for several weeks. Our previous study showed that a one-time freeze and thaw cycle of PLGA NPs from −20 °C did not affect the size of NPs, and PLGA can retain its stability in size up to four times in various studies [48,49,50]. However, multiple freeze-thaw cycles (more than four times) affected the stability of NPs in other studies [51,52]. In our previous studies, we showed that PLGA NPs are compatible with red blood cells and did not cause hemolysis across the dose we used in vitro and in vivo [19,20]. In this study, the release profile of DRV showed that at 48 h, ~50.2% of loaded DRV is released. We used the freshly prepared formulation within 48 h for in vitro and in vivo treatment. The EE% of the PLGA-DRV is ~84.19%, which is considered a good EE% for PLGA NP-encapsulated drugs using the nanoprecipitation method.

HIV infection is associated with a reduction in antioxidant defenses [53]. The virus and its proteins can interfere with the normal functioning of antioxidants, worsening the oxidative stress of the infected cells [53,54]. The increased oxidative stress in HIV-infected individuals can contribute to inflammation. HIV infection leads to oxidative stress by increasing the production of ROS [55]. ROS can activate various signaling pathways that lead to the production of pro-inflammatory cytokines, further fueling the inflammatory process [33]. Certain antiretroviral medications, like 2′,3′-dideoxycytidine, have the capability to cross the BBB [56]. However, they can induce oxidative stress in the brain, causing the induction of HIV dementia [56]. The increase in oxidative stress and the alteration in antioxidant enzymes in HIV-infected myeloid cells are key features of HIV. The ROS level was significantly decreased when U1 was treated by PLGA alone and PLGA-DRV. The findings suggest that PLGA-DRV is not only safe from an oxidative stress perspective, but it can also suppress HIV-induced oxidative stress in macrophages.

Cytokines play an important role in controlling the homeostasis of the immune system [57]. In our studies, we studied the ability of PLGA-DRV to affect HIV-related pro-inflammatory cytokines and chemokines. HIV infection in the CNS occurs when infected immune cells, such as monocytes and T cells, cross the BBB and carry the virus into the brain [58]. Due to the absence of necessary receptors for the virus, neurons are not directly infected by HIV [59]. However, the effects of HIV infection in the CNS are profound and can lead to a spectrum of neurological complications [60]. HIV infection triggers the immune system’s activation, which is evidenced by elevated levels of cytokines and chemokines in the plasma [61]. Inflammatory cytokines subsequently lead to HIV progression and HIV neuropathogenesis via complex and overlapping pathways [62]. Therefore, in this study, we used the chronically infected HIV promonocytic cell line to learn the cytokine and chemokine expression after treatment of PLGA NPs.

TNF-α showed an HIV inhibitory effect by inducing the secretion of RANTES and decreasing the expression of CC chemokine receptor 5 (CCR5), which is a co-receptor of HIV infection [63]. RANTES blocks the entry of R5 strains into cells through CCR5 by competitively binding to and causing the downregulation of CCR5 [64,65]. However, it also induces inflammation in the HIV-infected cells. We did not observe any significant change in TNF-α among treatment groups. However, the expression of RANTES was significantly higher in PLGA NPs alone and PLGA-DRV groups. It appears that synthetic PLGA NPs can also increase the pro-inflammatory chemokine, RANTES, in U1 macrophages.

In our previous study on EVs derived from CSC-exposed macrophages, we showed that packaging IL-1β contributed to the increase in the IL-1β in astrocytes and neuronal cells and further led to HIV neuropathogenesis [66]. In direct treatment in U1 and in the presence of a BBB, the expression of IL-1β is significantly lower in PLGA-DRV than in the control group, although the reduction was not more than with DRV alone. Increased IL-6 levels have been observed in the serum and plasma of HIV patients even under ART, and IL-6 was considered a stronger predictor of fatal events related to AIDS [61,67,68]. IL-6 was significantly reduced in U1 following direct treatment with DRV, PLGA NPs, and PLGA-DRV as compared to the control group. However, this reduction was not observed in the in vitro BBB model with PLGA NPs and PLGA-DRV. IL-8 can be used as one of the hallmarks of chronic inflammation in HIV patients at the beginning of the treatment to indicate the progression of HIV [69]. During HIV infection, IL-8 secretion is increased, and in turn, the increase in IL-8 may facilitate HIV in vitro [33,57]. However, we did not see an increase in HIV replication in PLGA and PLGA-DRV treatment. This may be explained by the finding that the change in IL-8 expression did not contribute to latent HIV progression when U1 cells were treated by PLGA-DRV.

The level of IL-18 protein was found to have a positive correlation with viral load and a negative correlation with the frequency of CD4+ T cells in the gut-associated lymphoid tissue (GALT) [70]. Individuals who progress in HIV have higher IL-18 levels in PBMCs and plasma compared to HIV controllers [70]. IL-18R is consistently and widely present in neuronal cells across the brain, and elevated IL-18 levels are correlated to CNS disorders [71]. As expected, DRV alone deceased IL-18 both in direct treatment and in the treatment with BBB. However, PLGA NPs also show a similar decrease in IL-18 and no further decrease with PLGA-DRV.

Both IL1-RA and IL-10 play a crucial role as an anti-inflammatory cytokine, possessing the ability to curb inflammatory and autoimmune disorders. Their function extends to protecting against the detrimental effects of inflammation and autoimmunity, which can be particularly beneficial in the context of HIV infection [72]. IL-10 appeared to be increased with PLGA-DRV in direct treatment of U1 macrophages, and IL-10 was significantly increased in PLGA NPs alone as compared to control in the presence of a BBB. PLGA NPs significantly increased the IL-1RA levels in U1 macrophages. These anti-inflammatory cytokines may mitigate the neurological damage and cognitive impairments associated with HIV.

Overall, PLGA-DRV did not aggravate the inflammatory status in U1 macrophages. PLGA NPs alone appear to show anti-inflammatory response in IL-1β, IL-6, and IL-18 in direct treatment. Taken together, PLGA NPs alone or with encapsulated DRV in direct treatments or in the presence of a BBB appear to improve the overall immune response in U1 macrophages. In general, synthetic NPs could illicit an inflammatory response [73]. However, in this case, the increase in some of the pro-inflammatory cytokines and chemokines did not aggravate the HIV progression. Further studies are needed to verify the observation, especially in animal models.

Compared to DRV alone, exposure to PLGA-DRV in direct treatment significantly increases the drug concentration in U1 macrophages, suggesting the role of PLGA in facilitating the permeability of DRV in the cells. However, due to technical limitations, we were not able to determine the intracellular concentration of DRV across the BBB at different time points over the period of treatment. Therefore, we studied the ability of PLGA-DRV to penetrate the BBB and increase drug concentration in mice brains.

Our previous findings have established that IN administration of DRV elevates its concentration in the brain more effectively than other routes of administration [21]. In the mice study, after dosing PLGA-DRV at a dose of 2.5 mg/kg, we observed an increase in the DRV concentration in the brain in the PLGA-DRV group with the IN injection. The overall brain drug concentration was higher via the IN route than the IV administration, especially for the PLGA-DRV group. These findings are consistent with our previous study, where compared to IV, IN improved the drug concentration in the brain as well as the brain-to-plasma ratio. IN also showed a higher brain concentration than IV at corresponding time points, and the overall drug exposure in plasma and peripheral organs, such as livers, was higher in IV.

There is a difference in the DRV concentrations in the plasma, peripheral organs, and brain using two different routes of administration. Following IV administration, DRV and PLGA-DRV circulate within the bloodstream and are distributed to various peripheral organs, while some of the DRV and PLGA-DRV cross the BBB into the brain. The IV administration typically results in systemic drug exposure, leading to higher drug concentrations in the plasma and liver. In contrast, with IN administration, a portion of DRV is taken up by the olfactory bulb and directly accesses the brain via neurons in the nasal mucosa, while another portion is absorbed into the bloodstream [29,74]. Subsequently, in the IN route, DRV or PLGA-DRV partially cross the BBB in a manner similar to the IV route. This latter process of absorption via BBB permeability may require more time, which accounts for the observed peak in DRV levels at a later time point in the IN group.

Although it is challenging to dose mice through the IN route at a relatively high dose, the IN route appears to be an ideal administration route to improve drug delivery in the brain with lower peripheral drug exposure. It also requires a relatively low drug dose, which would further reduce off-target effects. Achieving and maintaining an effective concentration of DRV in the brain is crucial for maximal suppression of HIV, thereby addressing HAND. We are in the process of developing a relevant HIV mice model to examine the efficacy of PLGA-DRV on neuropathogenesis and HAND.

This study has the following limitations: (1) U1 is a promonocytic cell line derived from U937 macrophages, which may not fully replicate the behavior of HIV-infected macrophages in brain reservoirs. Therefore, a relevant HIV mouse model is needed in the follow-up study to validate the effect of PLGA-DRV on suppressing HIV replication, oxidative stress, and inflammation in CNS reservoirs. (2) DRV activity in terms of HIV replication and inflammatory response with PLGA-DRV appears to be the same as DRV alone across the BBB in vitro model. This could be because the BBB model is not optimal. Therefore, we performed an in vivo experiment with wild-type mice, which showed improved brain drug concentration with PLGA nanoformulation. Further, an in vivo study using an HIV mice model is important to determine whether PLGA-DRV can effectively suppress HIV neuropathogenesis. (3) Compared to DRV alone, PLGA-DRV did not significantly alter the inflammatory response in macrophages. The level of cytokines is known to vary at different stages of HIV. Huang et al. have shown that cytokine storms occurred on different days after HIV infection between rapid disease progressors and slow disease progressors [75]. This may explain our in vitro study, which was based on acute treatments up to 48 h. It is difficult to perform a chronic experiment with differentiated U1 macrophages with or without BBB in vitro models.

## 5. Conclusions

To the best of our knowledge, this is the first study to show that PLGA-DRV can suppress HIV replication, oxidative stress, and inflammatory response without causing cytotoxicity in U1 macrophages directly and across the in vitro BBB models. Although PLGA-DRV does not further decrease HIV replication and inflammatory response compared to DRV alone, at least it retains the effects both in direct treatment and across the BBB. This is also the first study that showed improved DRV permeability by PLGA nanoformulation in macrophages and in the brains of wild-type mice, especially using the IN route. The PLGA-DRV nanoformulation also reduced DRV exposure to peripheral organs using the IN route. The IN administration using PLGA-encapsulated drugs would not only increase drug efficacy in the brain, but it could also reduce off-target effects in peripheral organs. This study is the first step to developing PLGA-encapsulated ART drugs, especially using the IN route, into therapeutics to suppress neuroHIV and HAND. The next goal of this study is to apply this drug delivery system to HIV mice and study the safety and efficacy, including cognitive functions that have the potential to prevent/treat HAND. Although PLGA is an FDA-approved NP, we will also explore other natural biocompatible and targeted nanoparticles for the treatment of HIV in the brain, which are biodegradable and have no/tolerable toxicity and immune response in the brain [37,76].

## Data Availability

The raw data supporting the conclusions of this article will be made available by the authors without undue reservation.
